# Perineuronal Nets Play a Role in Regulating Striatal Function in the Mouse

**DOI:** 10.1371/journal.pone.0032747

**Published:** 2012-03-12

**Authors:** Hyunchul Lee, Catherine A. Leamey, Atomu Sawatari

**Affiliations:** Discipline of Physiology, School of Medical Sciences and the Bosch Institute, University of Sydney, Sydney, Australia; University of Chicago, United States of America

## Abstract

The striatum is the primary input nucleus of the basal ganglia, a collection of nuclei that play important roles in motor control and associative learning. We have previously reported that perineuronal nets (PNNs), aggregations of chondroitin-sulfate proteoglycans (CSPGs), form in the matrix compartment of the mouse striatum during the second postnatal week. This period overlaps with important developmental changes, including the attainment of an adult-like gait. Here, we investigate the identity of the cells encapsulated by PNNs, characterize their topographical distribution and determine their function by assessing the impact of enzymatic digestion of PNNs on two striatum-dependent behaviors: ambulation and goal-directed spatial learning. We show PNNs are more numerous caudally, and that a substantial fraction (41%) of these structures surrounds parvalbumin positive (PV+) interneurons, while approximately 51% of PV+ cells are ensheathed by PNNs. The colocalization of these structures is greatest in dorsal, lateral and caudal regions of the striatum. Bilateral digestion of striatal PNNs led to an increase in both the width and variability of hind limb gait. Intriguingly, this also resulted in an improvement in the acquisition rate of the Morris water maze. Together, these data show that PNNs are associated with specific elements of striatal circuits and play a key role in regulating the function of this important structure in the mouse.

## Introduction

The striatum, the primary input nucleus of the basal ganglia, is important in motor control and associative learning [1], [2], [3], [4], [5]. This notion is reinforced by motor, cognitive and motivational deficits manifested in striatal disorders such as Parkinson's disease, Huntington's chorea, and autoactivation disorder [6], [7], [8], [9]. Plasticity, the ability of neural circuits to adapt to changing extrinsic and intrinsic conditions, is thought to play a central role in striatal function [5], [10], [11].

Rapid, dramatic changes in plasticity across entire brain regions are a hallmark of neural development. For example, in sensory cortex, there exists a ‘critical period’ during which circuits become particularly sensitive to environmental/activity dependent manipulation [12], [13], [14], [15]. Moreover, the timing of this developmental window itself can be influenced by the animal's early experiences and perinatal/neonatal surroundings [16], [17].

The balance between excitatory and inhibitory activity is considered crucial for the onset and offset of cortical critical periods. The timing of these epochs has been shown to be dependent on the physiological ‘maturation’ of inhibitory networks consisting of parvalbumin-positive (PV+), fast-spiking interneurons (reviewed in [14], [18]). This event is concurrent with the appearance of perineuronal nets (PNNs), extracellular matrix structures rich in chondroitin sulfate proteoglycans (CSPGs) and associated with PV+ cells. These structures are not only considered important in consolidating synaptic connections [17], [19], [20], they may also modulate the excitatory-inhibitory balance by influencing the activity of inhibitory interneurons [21], [22]. Crucially, the enzymatic removal of PNNs by chondroitinase ABC (ChABC) can re-establish critical period-like plasticity in primary visual cortex [23], as well as in non cortical areas like the basolateral amygdala [24].

We recently revealed that PNNs appear in the striatal matrix during the second postnatal week in the developing mouse, coincident with the maturation of PV+ interneurons within the striatum and the transition from a forelimb dependent ‘crawl’ to a more mature gait in the ambulation of juvenile pups [25]. Matrix medium spiny neurons (MSNs), a major downstream target of PV+ cells, also exhibit consistent and significant changes in dendritic morphology during this same period [26]. Further, environmental enrichment can accelerate both the onset of striatal PNN formation as well as the emergence of exploratory and coordinated motor behavior [27]. All these observations point to the possibility that this striatal postnatal epoch is analogous to the ‘critical period’ as described in sensory cortex, the time course of which is dependent on excitatory-inhibitory balance. If PNNs play a similar role in regulating the maturation and plasticity of circuits within the striatum, then there should be a detectable relationship between striatal PNNs and PV+ cells, as well as functional changes when these structures are removed.

In order to address these issues, we show using combined immunohistochemistry and lectin-binding, that striatal PNNs are associated with PV+ neurons in a topographically specific manner. Further, by employing behavioral assays we provide evidence of alterations in striatal function. Selective removal of striatal PNNs via ChABC treatment causes widening and increased variability in gait, as well as affecting the acquisition of the Morris water maze (MWM). These results suggest that PNNs may play an important role in the regulation of striatal function.

## Results

### Striatal parvalbumin-positive cells are associated, but not exclusively, with perineuronal nets

**Figure 1:**
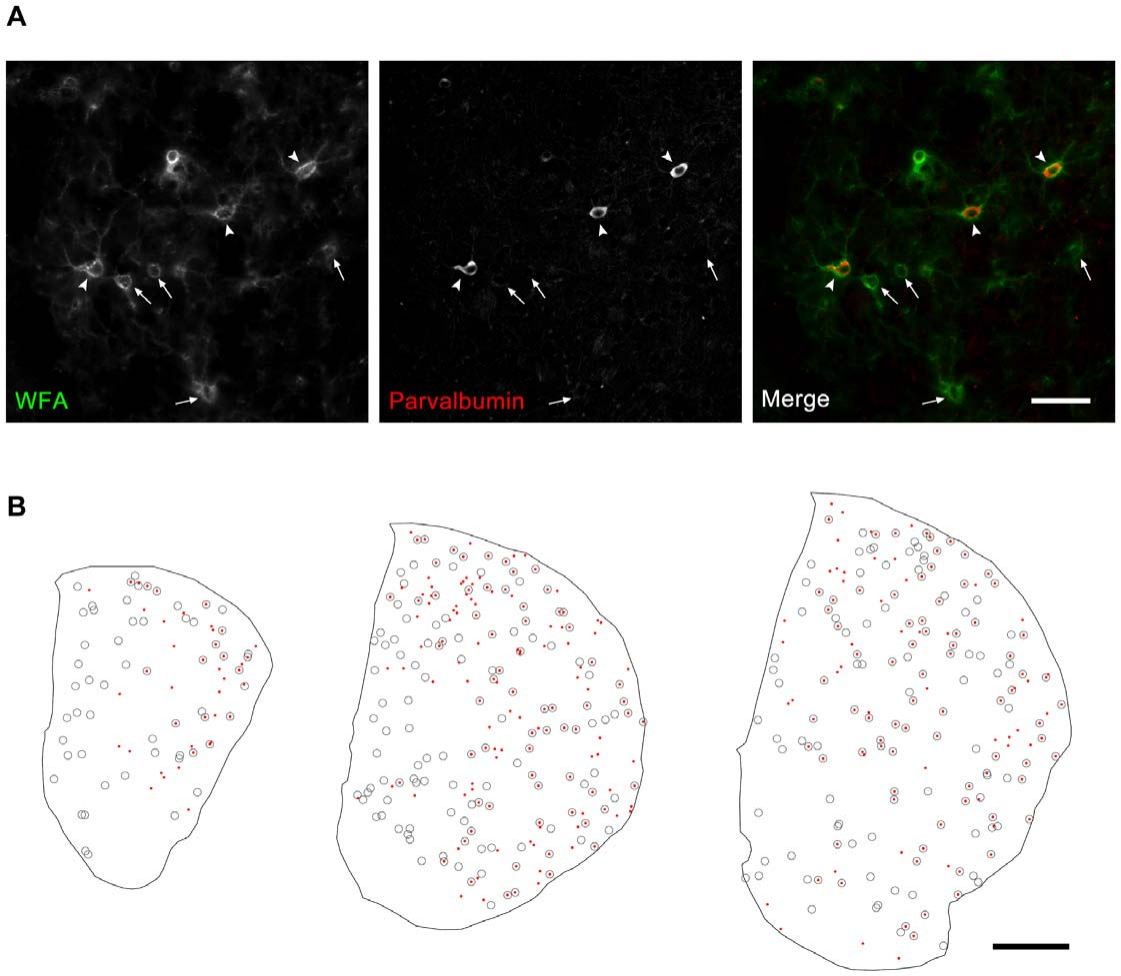
PNNs show considerable, but not exclusive overlap with PV+ cells in the striatum. (A) Panels show high power micrographs revealing WFA/PNN labeling (left), PV+ immuno-staining (middle), and a merged image of the two (right). Although WFA/PNN and PV+ signals overlapped considerably (arrowheads), both PNNs without PV+ co-labeling (arrows), as well as PV+ cells without PNNs (not visible in this section) were also present. Scale bar: 100 µm. (B) Sample reconstructions of three coronal sections from the anterior striatum (Left to right: rostral, middle, and caudal) showing locations of PV+ cell bodies (red dots) and PNNs (grey rings). Both the degree as well as localization of PNN/PV+ overlap can be seen to vary across the rostro-caudal axis. Right: lateral, up: dorsal. Scale: 500 µm.

In order to determine whether striatal PNNs are associated specifically with PV+ interneurons, double labeling of *Wisteria floribunda* agglutinin (WFA) lectin-binding and parvalbumin immunohistochemistry was performed on sections of mouse striatum. Although many PNNs were observed to surround the soma and proximal dendrites of PV+ cells (arrowheads, Fig. 1A), the correspondence between these structures was not absolute (arrows, Fig. 1A). In order to quantify the distribution of both these structures, as well as their degree of overlap topographically, comparisons from reconstructions were made across rostrocaudal, mediolateral, and dorsoventral axes (sample reconstructions shown in Fig. 1B).

**Figure 2:**
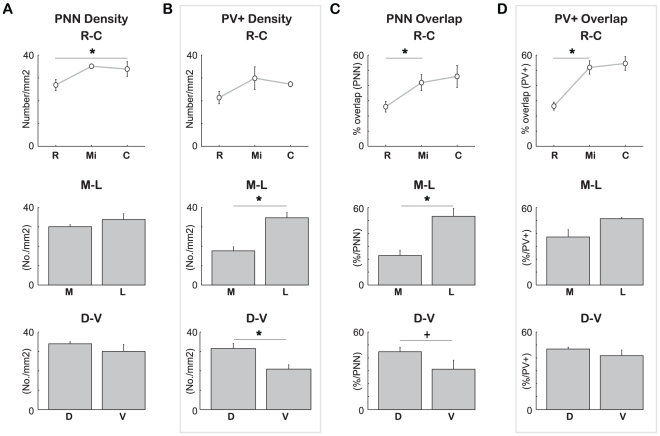
PNN/PV+ cell density as well as overlap depend on topographic location within the striatum. (A) Rostral striatum exhibited a slightly decreased density of PNNs compared to the caudal most regions (pairwise comparisons, Bonferroni corrected; *P* = 0.039, top panel). No differences were observed along either the mediolateral (middle panel) or dorsoventral axes (bottom panel). (B) In contrast, no difference was seen along the rostrocaudal axis in PV+ density (top panel). Highly significant differences, however, were observed across both mediolateral (RMANOVA; *P* = 0.002, middle panel), and dorsoventral axes (RMANOVA; *P* = 0.016, bottom panel), reflective of distributions observed in other species. (C) A significant difference in the percentage of PNNs associated with PV+ cells was observed in the rostral-caudal axis, with the rostral most section exhibiting significantly less overlap compared to the middle section (RMANOVA; within subject contrasts for sections, *P* = 0.033., top panel). Significant differences were also observed in mediolateral axis, with medial striatum showing highly significantly less association between these two structures (RMANOVA; *P* = 0.005, middle panel). Although overlap was less in ventral vs. dorsal regions, this difference did not reach significance (RMANOVA; *P* = 0.068, bottom panel). (D) Similarly, rostral sections show a significantly smaller percentage of parvalbumin-positive cells surrounded by PNNs compared to more caudal areas of the striatum (RMANOVA; within subject contrasts for section, *P* = 0.036, top panel). No differences were observed in the other axes (middle and bottom panels). R: rostral; Mi: middle; C: caudal; M: medial; L: lateral; D: dorsal; V: ventral. *: *P*<0.02. +: *P*<0.07.

PNNs were present throughout the striatum, with what appeared to be a slight bias towards dorsal regions. Large gaps in their distribution were observed, consistent with their relative avoidance of striosomes [25]. A small effect was detected across the rostrocaudal axis (number/mm^2^, mean ± standard error: rostral (R): 26.897±2.413; middle (Mi): 35.104±0.94; caudal (C): 33.889±3.221; RMANOVA, *F*(2,4) = 7.992, *P* = 0.04; Fig. 2A, top panel): pairwise comparisons yielded a significant difference between the most rostral and caudal sections assessed (*P* = 0.039, Bonferroni corrected). No significant differences across mediolateral (medial (M): 30.133±1.038; lateral (L): 33.794±3.022; *F*(1,2) = 1.192, *P* = 0.389; Fig. 2A, middle panel) or dorsoventral axes (dorsal (D): 33.944±1.137; ventral (V): 29.983±3.659; *F*(1,2) = 3.191, *P* = 0.216; Fig. 2A bottom panel) were observed.

For PV+ cells, no difference was observed across the rostrocaudal axis (number/mm^2^, mean ± standard error: R: 21.358±2.679; Mi: 29.865±5.002; C: 27.264±1.124; RMANOVA, *F*(2,4) = 2.255, *P* = 0.221; Fig. 2B, top panel). Across both mediolateral and dorsoventral axes, however, the density of these neurons varied considerably, with significantly higher concentrations seen in lateral (L: 34.702±2.649; M: 17.623±2.096; *F*(1,2) = 426.281, *P* = 0.002; Fig. 2B, middle panel) and dorsal (D: 31.472±2.626; V: 20.853±2.254; *F*(1,2) = 62.115, *P* = 0.016; Fig. 2B, bottom panel) regions of the striatum. A significant interaction was also observed between rostrocaudal and mediolateral axes (*F*(2,4) = 8.930, *P* = 0.033). These findings are consistent with previously described distributions of PV+ interneurons within the striatum [28], [29].

A relatively high proportion of PNNs were clearly associated with PV+ interneurons (40.8±10.6%). As these interneurons comprise only 0.7% of the striatal population [28], the level of overlap was much higher than would be expected by chance (one tailed t-test, *P* = 0.023). Similarly, approximately half of the labeled PV+ cells were encapsulated by PNNs (50.8±2.0%).

Moreover, the degree of association between PNNs and PV+ cells exhibited significant differences topographically across the striatum. Along the rostrocaudal axis, the proportion of PNNs that were associated with PV+ interneurons differed (RMANOVA, *F*(2,4) = 20.604, *P* = 0.008) with the rostral-most section having significantly less overlap (% overlap, mean ± standard error: R: 26.1±3.5; Mi: 41.9±5.2; C: 46.0±7.2; within subject contrast between rostral and middle sections, *P*  =  0.033; Fig. 2C, top panel). A significantly greater association was also observed in lateral relative to medial regions of the striatum (L: 53.2±6.2; M: 22.8±4.1; *F*(1,2) = 214.008, *P* = 0.005; Fig. 2C, middle panel). A similar though non-significant trend was also observed across the dorsoventral axis, with ventral regions exhibiting less overlap compared to dorsal areas (D: 44.8±3.4; V: 31.2±7.0; *F*(1,2) = 13.307, *P* = 0.068; Fig. 2C, bottom panel).

A similar pattern was observed when considering the proportion of PV+ cells surrounded by PNNs. Here too, differences were observed along the rostrocaudal axis (*F*(,2, 4) = 17.546, *P* = 0.010), with the rostral-most region showing significantly less overlap (% overlap, mean ± standard error: R: 26.4±2.6; Mi: 51.9±4.4; C: 54.5±4.6; within subject contrast between rostral and middle sections, *P* = 0.036; Fig. 2D, top panel). Curiously, however, no significant differences were observed for the other two axes (Fig. 2D, middle and bottom panels).

### Striatal perineuronal nets are not associated with cholinergic or calretinin positive interneurons

In order to further determine the identity of PV− cells associated with striatal PNNs, double labeling for WFA and two other proteins associated with different interneuron subtypes were performed: choline acetyltransferase (ChAT), a marker for large cholinergic interneurons, and calretinin (Cr), a Ca^2+^ binding protein associated with a third non-projecting group of neurons within the striatum.

Interestingly neither cell type exhibited any association with PNNs (Fig. S1). Although the large ChAT positive cells were easy to identify, none were observed to be surrounded by WFA positive PNNs anywhere within the striatum (Fig. S1A). Similarly, the smaller Cr+ cells also showed no correspondence with PNN encapsulated cells (Fig. S1B). Thus, the relatively large number of PNN associated PV− cells must comprise other cell types, possibly MSNs or somatostatin positive interneurons.

### ChABC treatment removes PNNs for over two weeks in the striata of adult mice

PNNs have been shown to regulate the plasticity of neural circuits important for sensory function and fear conditioning [23], [24]. To determine whether PNNs play a similar role in behaviors associated with striatal function, we removed these structures using chondroitinase ABC (ChABC). This enzyme digests chondroitin sulfate proteoglycans (CSPGs), the main constituents of PNNs. Previous studies have revealed that PNNs reform after enzymatic digestion, in an age-dependent manner, with recovery slower in older animals [30]. Accordingly, in order to determine whether striatal PNNs remain absent during the period required for behavioral testing, the striata of 8+ months old adult mice injected unilaterally with ChABC were assessed for WFA labeling at two different time points (3 and 14 days post-injection), flanking the entire task training and assessment period. PNNs were completely removed from the treated region after 3 days, with little recovery of these structures in the injected hemisphere even after two weeks (Fig. S2). Importantly, PNNs were present in the cortex and other structures surrounding the striatum in the injected hemisphere, indicating that any functional effects of this treatment are likely to be specific to the striatum. In order to confirm that PNNs remained digested throughout the behavioral testing and training periods, WFA labeling was performed on the striata of all animals immediately after assessment (see Methods, and below).

### Removal of striatal PNNs leads to increased width and variability of hind limb gait

**Figure 3:**
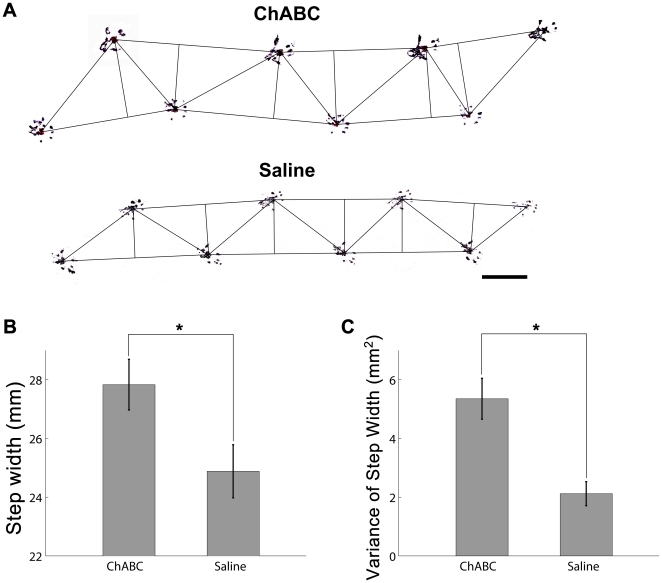
Striatal ChABC treatment significantly increases step width and variance in adult mice. Sample pawprints from ChABC (top) and vehicle (bottom) treated mice (A; see Methods for measurement details). Scale bar: 20 mm. (B) Bar graph showing mean ± standard error of step width for mice receiving either ChABC (ChABC) or vehicle (Saline) injections in bilateral striatum. Enzyme treated animals exhibited significantly increased hind limb gait widths relative to saline controls (Wilcoxon rank sum test; *P* = 0.0276). *: *P*<0.05. (C) Variance, the variability in measured step width for a given subject, was also significantly greater in ChABC treated subjects (Wilcoxon rank sum test; *P* = 0.003). *: *P*<0.05.

PNN formation within the postnatal developing striatum coincides with a transition from an immature, predominantly forelimb dependent ‘crawl’ to a more mature gait in mice [25]. This tight temporal relationship suggests that PNNs may play a vital role in consolidating the circuits necessary for the control of adult-like limb movements. In order to assess this possibility, we compared hind limb step width and variability in mice that were treated with either ChABC or vehicle injections in bilateral striatum. Remarkably, enzyme-treated mice exhibited both a significantly larger mean step width (Wilcoxon's rank sum test, *P* = 0.0276; Fig. 3A, B), as well as a greater step width variability (Wilcoxon's rank sum test, *P*<0.0030; Fig. 3A, C) compared to saline controls. This indicates that the targeted removal of striatal PNNs can measurably affect the hind limb gait of mice.

### Removal of striatal PNNs leads to improved Morris water maze acquisition

Although the effect of ChABC treatment on gait width is consistent with striatal PNNs playing a role in motor control, the striatum itself is also involved in the regulation of other functions, particularly in the establishment of contextual and/or goal directed learning. It is thus possible that the digestion of striatal PNNs may lead to other, non-motor changes in behavior. In order to test this explicitly, we made a preliminary assessment of ChABC and vehicle-treated adult mice on a single submerged platform version of the MWM with external cues. Previous studies have shown that focal bilateral striatal lesions can lead to significant deficits in the acquisition of this task [31], [32].

**Figure 4:**
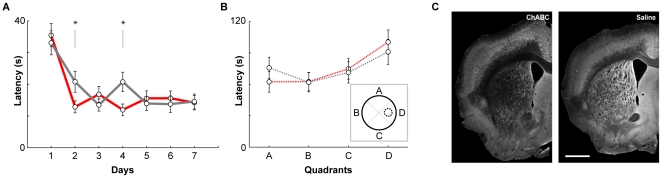
Removal of striatal PNNs improves Morris water maze acquisition. A significant interaction between treatment Group and training Days was observed in terms of latency to goal (Repeated measures ANOVA, group versus days interaction, *P* = 0.013) (A). Closer analysis revealed that ChABC treated animals (red) took significantly shorter amounts of time to find the submerged platform on Days 2 and 4 (ANOVA, P<0.05). (B) Although both ChABC (red dotted) and vehicle (grey dotted) treated groups exhibited a preference for the quadrant that previously contained the submerged platform (Quadrant D; inset), no significant difference was observed between the two groups. (C) Sample hemispheres of ChABC (left) and saline (right) treated striatum from animals that had completed behavioral testing. PNNs remained digested even after two weeks of training. Scale bar: 1 mm.

Remarkably, a significant interaction was observed between treatment groups and days training for latency (RMANOVA, days training by group interaction, *F*(6,11) = 4.733, *P* = 0.013; Fig. 4A), as well as distance travelled (RMANOVA, days training by group interaction, *F*(6,11) = 3.197, *P* = 0.045) indicating that the acquisition of the task was slightly improved in ChABC treated mice. No main effect between groups was observed for either measurement, suggesting the modest effect was limited to the rate of acquisition, and not the ability to acquire the platform location per se (between subject comparisons for groups: latency: *F*(1,16) = 0.219, *P* = 0.646; distance: *F*(1,16) = 0.312, *P* = 0.584; see below). The average swim speed did not differ between the groups (days training by group interaction: *F*(6, 11) = 2.219; *P* = 0.119; main effect for groups: *F*(1,16) = 0.312, *P* = 0.584). Comparison of performances between groups for each block of trials per day (Day) revealed that ChABC treated mice exhibited significantly decreased latencies on Day 2 and Day 4 (One way ANOVA comparison of groups; Day 2: *F*(1,70) = 4.294; *P* = 0.042; Day 4: *F*(1,70) = 0.015). Similarly, treated animals swam for shorter distances on Day 4 (One way ANOVA comparison between groups; Day 4: *F*(1,70) = 7.175; *P* = 0.009). Differences between groups were not present in any of the measurements for all other Days. Together, these findings indicate that the slight improvement in platform acquisition exhibited by ChABC treated mice were not due to changes in motor ability.

In order to further determine whether PNN digestion affected maintenance of place acquisition, ChABC and vehicle treated mice were tested on a single probe trial in which the submerged escape platform was removed. Although both cohorts exhibited a bias (in terms of time spent) for the quadrant which previously contained the platform (Quadrant D, inset, Fig. 4B), no difference was observed between the two groups (RMANOVA, group vs. sector, *F*(3, 14) = 0.462, *P* = 0.713), indicating that the ability to maintain and recall the goal location was not compromised in ChABC treated mice (Fig. 4B).

Post training examination confirmed that PNNs remained sparse immediately after completion of behavioral experiments relative to saline controls (Fig. 4C). In order to determine the extent of PNN digestion, the affected region for three representative ChABC treated animals was measured as a percentage of total striatal area. On average, 36.3±8.5% (mean ± standard deviation) of both hemispheres (n = 5 hemispheres) remained PNN free for these animals.

## Discussion

This study provides, to the best of our knowledge, the first quantitative assessment of PV+ interneuron distributions within the adult mouse striatum. We reveal a strong, but not exclusive, association between striatal PNNs and this cell type in a manner which varies topographically. Further, in order to determine a possible role for PNNs in striatal circuits, we tested animals that received bilateral striatal injections of ChABC for their hind limb gait and performance on the MWM. We found that enzymatic removal of striatal PNNs altered gait width and variance. Moreover, ChABC treated mice exhibited a slight but significant enhancement in MWM acquisition. These findings indicate that PNNs play a vital role in regulating striatal function.

### PNNs and PV+ interneurons exhibit different topographic density distributions within the mouse striatum

Striatal PNN densities were slightly, but significantly, lower in rostral compared to the most caudal section analyzed. We have previously reported that striatal PNNs exhibit a strong preference for the matrix compartment [25]. Given that striosomes in rodent striatum exhibit a high rostral to low caudal gradient [33], [34], the bias we see in these CSPG structures may be due to the skewed distribution of striatal subcompartments along this topographical axis.

PV+ cells also exhibited differences in densities topographically: concentrations of these neurons were significantly greater in dorsal and lateral regions of the striatum. This patterning is similar to previously described striatal PV+ distributions in rodents [28], [29], [35], as well as known regional differences in striatal connectivity and function [36], [37].

### PNNs target PV+ as well as PV− neurons in the mouse striatum

To the best of our knowledge this is the first report describing a preferential association of PNNs with PV+ cells in the adult mouse striatum. The observed overlap was significantly greater than chance, as these fast-spiking GABAergic interneurons make up only 0.7% of the striatal cell population [28]. This finding is consistent with the robust colocalization of PNNs with PV+ fast-spiking interneurons described in other brain areas [20], [38].

We also observed, however, a large number of PNNs that were not associated with striatal PV+ cells. Although other cell types are known to be ensheathed by PNNs in other brain areas [39], [40], the extent of PV negative (PV−) cells associated with PNNs in this important regulatory structure was surprising. Preliminary results indicate that neither ChAT nor Cr+ interneurons are associated with these CSPG structures. These observations suggest that the most likely candidates for these PV− cells are either somatostatin positive interneurons, or a subpopulation of MSNs. Studies are currently underway to assess this possibility.

Afferent activity has been shown to contribute to the formation of cortical PNNs [13], [21], [23], [41]. Given these observations, it is intriguing that the localization of cell types associated with striatal PNNs differ topographically in a manner consistent with known cortical/subcortical input. Previous studies in the cortex have shown that even in areas or layers of overlapping cellular populations, individual neuronal cell types can receive different, yet consistent local excitatory [42] as well as inhibitory [43] input. Dorsal and lateral striatum receives input from sensory and motor areas of the cerebral cortex, a considerable proportion of which terminates on PV+ interneurons [36]. It is possible that the relatively high number of PNNs associated with PV+ cells in these regions reflect the convergent excitatory drive experienced by this particular cell type.

On the other hand, we observed that most PNNs in ventral and medial striatum surround PV− cells. As these regions are targeted by a host of different limbic and subcortical structures [44], [45], our findings raise the intriguing possibility that specific, convergent input from such areas may also contribute to PNN formation on cell types other than PV+ interneurons. Further studies focusing on the specificity and consistency of input to individual cell types associated with striatal PNNs will be required to determine if this is the case.

### Striatal PNNs play a crucial role in consolidating hind limb gait

In order to further determine a possible role of PNNs in the function of adult striatal circuits, we assessed the performance of mice receiving bilateral ChABC injections in the striatum on hind limb gait, a measure of coordinated motor behavior. Interestingly, the specific, targeted digestion of striatal PNNs led to mice exhibiting wider and more variable hind limb placements. Potential lesions to the striatum caused by our surgical procedure cannot account for these findings, as the differences were observed over vehicle controls which were handled and treated identically.

The types of gait changes observed here following digestion of striatal PNNs resemble limb movement and coordination deficits previously reported in mice that have received 6-OHDA injections within the substantia nigra pars compacta (SNc) [46], [47]. It is somewhat surprising that a relatively subtle manipulation such as the enzymatic removal of an extracellular matrix structure within the striatum can have as dramatic an effect on hind limb gait as that resulting from the removal a major source of DA afferents. CSPGs can act as a chemoaffinity signal for DA projections during early development [48]. Moreover, the activity of PV+ cells is modulated by DA [49], and PNNs are involved in stabilizing synapses terminating onto cells ensheathed by these structures. Further, striatal PNNs begin forming in the matrix compartment between the first and second postnatal weeks [25], roughly the time when DA afferents begin to terminate within this subregion, and when the “broad-based and wobbly” gait of juvenile rodents (limbs generally spread distally and variably from the body center) transitions to a more adult like ambulation (limbs assuming a more vertical position under the body) [50], [51]. It is therefore possible that the changes observed in hind limb coordination may in part be due to a ‘decoupling’ of DA afferents specifically from PNN associated neurons as a result of ChABC digestion.

Given the close temporal correlation between the appearance of the PNNs and the attainment of adult-like gait, and our evidence that digestion of these structures leads to a variable walking pattern reminiscent of more immature animals, it is tempting to speculate that the formation of PNNs is critical for the functional maturation of circuits that underlie this sensorimotor behavior. This is consistent with recent work which has shown that the formation of these same CSPG structures in a key pre-motor forebrain structure correlate with the acquisition of song in songbirds [52]. Here too, the maturation of this ethologically vital sensorimotor behavior is characterized by a transition from highly variable vocalizations to a more stereotyped, ‘crystallized’ song (reviewed in [53]).

### Striatal PNNs regulate the acquisition of a goal-directed spatial learning task

The removal of PNNs led to a slight but significant improvement in the acquisition of the Morris water maze. Since the most marked change was a decrease in latency in ChABC treated mice, reduced swimming ability cannot account for these results. This lack of a decrease in swimming ability may seem somewhat at odds with the observed differences in hind limb gait. Although both motor activities require coordinated, rhythmic movements, cutaneous and proprioceptive feedback differs considerably when engaged in these two actions [54]. Moreover, previous work has shown that although lesions to the motor pathway can influence both means of locomotion, the degree to which they are affected can vary [55], [56]. Thus, although further studies will be required to address this issue explicitly, it remains possible that enzymatic digestion of PNNs may have a differential effect on walking versus swimming behavior.

Both ChABC and vehicle treated groups were able to acquire the location of the platform by the end of the training period. This convergence was not due to PNN regrowth, as these structures remained digested in the striatum throughout the extent of the task. The lack of difference observed in the probe test further corroborates the notion that ChABC treatment did not compromise place learning *per se*.

Previous studies have shown that animals with striatal lesions exhibit deficits in behaviors that require the formation of cue-outcome associations [57]. Further, removal of the dorsomedial striatum can delay, but not prevent, platform acquisition in the MWM [31], [32]. This is in contrast to animals that have had lesions to hippocampal related structures [32], [58], which show a persistent inability to acquire the platform location. Our findings are therefore consistent with the notion that the caudate/putamen and hippocampus provide different, but potentially complimentary contributions in spatial learning tasks.

In light of this, it is curious that the removal of PNNs lead to a slight improvement in the rate of platform acquisition. Rodents with lesions to the caudate/putamen can show either no deficit, or under specific reinforcement conditions (e.g. the presentation of redundant stimulus cues), exhibit an enhancement in performance of an instrumental learning task [59]. In our MWM protocol, however, no extra reinforcement cues were used. Moreover, as stated above, potential lesions to the striatum caused by our surgeries cannot account for the changes in performance observed here as improvements were detected over vehicle controls, indicating that these differences are specifically associated with PNN digestion. To the best of our knowledge, improvement in MWM performance under conditions similar to those used here has not been previously reported in animals experiencing striatal lesions.

Interestingly, rats have been shown to exhibit significantly better performance across the first couple of trials at postnatal (P)22 compared to when they were retested at age P100 on a modified MWM [60], suggesting that spatial learning may be faster in younger animals. The degree to which the striatum contributes to this difference, however, has not been established. Although our findings provide support for the intriguing possibility that the removal of striatal PNNs leads to behavior reflective of a more immature state of the animal, further studies will be required to test the validity of this hypothesis.

The importance of PNN-constituents in synaptic plasticity and neuronal development is well established [61], [62], [63], [64]. In this context, a previous study has demonstrated that the removal of PNNs by ChABC in the primary visual cortex of adult rats reinstates ocular dominance plasticity normally found in younger animals [23]. As PNNs reflect the maturation of inhibitory networks, these structures are thought to play a crucial role in defining cortical critical periods by modulating the emergent balance between excitatory and inhibitory activity that is necessary for cortical plasticity [14], [18]. The reversion of fear memory to a form susceptible to extinction normally specific to younger animals, resulting from the removal of PNNs in the basolateral amygdala, may be due to similar mechanisms [24].

Our findings are consistent with a similar role for these structures in striatum. Striatal PNNs are first observed during the second postnatal week, coincident with the emergence of exploratory behavior and coordinated movements [27]. Moreover, we have now shown that digesting these striatal CSPG structures leads to changes in behavior that suggest increased network-level plasticity. Further studies are required to determine whether removal of striatal PNNs lead to changes in axonal and/or dendritic arborization or synaptic efficacy. Nevertheless, our findings provide evidence that these CSPG structures play a key role in regulating the function of circuits within the striatum.

## Methods

### Ethics Statement

Adult male C57BL/6J mice were used in these experiments. Animals were housed in a single adequately-ventilated room in 21°C ambient temperature on a 12-hour light-dark cycle with *ad libitum* access to dry food and water. All procedures were approved by the Animal Ethics Committee of the University of Sydney (AEC approval No.: K22/6-2006/3/4348 and K22/9-2009/3/5128) and conformed to National Health and Medical Research Council of Australia guidelines.

### Lectin binding/immunohistochemistry

#### Lectin binding to visualize PNNs

Mice were euthanized with an overdose of Euthal (sodium pentobarbital), then transcardially perfused with normal saline, followed by cold 4% paraformaldehyde (4°C). Brains were removed, postfixed overnight, then cryoprotected in 30% sucrose solution before being embedded in gelatin-albumin and sectioned coronally at 60 µm thickness on a freezing microtome (Leitz, Germany). Sections were collected in 0.1 M phosphate buffer (PB) then processed for lectin-binding as described previously [25]. Briefly, free-floating sections were washed in PB before endogenous peroxidases were quenched in a mixture of 45% (v/v) ethanol and 0.3% hydrogen peroxide (in PB) for 30 minutes. After washes in PB, sections were incubated in 10 µg/mL biontinylated *Wisteria floribunda* agglutinin (WFA, Vector Labs, Burlingame, CA, USA) for 2 hours at room temperature to label PNNs. Bound lectin was then conjugated to horseradish peroxidase using an ABC kit (Vector Labs) and visualized with fluorescein via tyramide signal amplification (Perkin Elmer, Waltham, MA, USA). Sections were then washed and mounted on gelatin-coated slides. After dehydrating in increasing concentrations of ethanol, slides were coverslipped with Entellan (EMS, Hatfield, CT, USA) and air dried.

#### Immunohistochemistry

Striatal sections were acquired from adult animals (1 month+ in age) that had not experienced ChABC treatment or behavioral assessment (see below). Briefly, WFA-labeled sections were washed in 0.3% Triton-X100 in PB before being incubated in a mixture of either rabbit anti-parvalbumin polyclonal antibody (1∶2000; Abcam, Cambridge, MA, USA) and 2% normal goat serum (Vector Labs) (n = 3 animals), rabbit anti-calretinin polyclonal antibody (1∶500, Abcam) and 2% normal goat serum (Vector Labs) (n = 2 animals), or goat anti-choline acetyl transferase polyclonal antibody (1∶500, Millipore, Billerica, MA, USA) and 2% normal rabbit serum (n = 2 animals) in PB overnight at room temperature. Sections were re-washed, then incubated in Texas-red conjugated goat anti-rabbit polyclonal antibody (rabbit anti-goat polyclonal antibody for anti-ChAT) (Invitrogen, Carlsbad, CA, USA) in 0.3% Triton-X100 and 2% normal goat serum (2% normal rabbit serum for anti-ChAT) for 2 hours before being washed and mounted on gelatin-coated slides. Slides were dehydrated, coverslipped, then air dried as above.

### Mapping PNNs/PV immunoreactive cells

Images of sections double-labeled with WFA and PV immunohistochemistry were acquired using a Zeiss LSM510 META confocal microscope with either 10× (N.A. 0.8) or 20× (N.A. 0.45) objectives.

To analyze the distribution and extent of overlap between PNNs and PV+ cells within the striatum, reconstructions with positions of each of these structures recorded separately were generated using Adobe Photoshop (CS3 extended) from three coronal sections of the striatum (rostral (R), middle (Mi), and caudal (C), sampled at distances roughly 240 µm apart) anterior to the anterior commissure. The reconstructions were then overlaid to generate a single PNN/PV+ ‘map’. PNN/PV+ cells were counted and the positional coordinates acquired on Cartesian axes using ImageJ 1.40 g (National Institutes of Health). Measurements were then imported into MATLAB (version 7.1; Mathworks, Natick, MA, USA) or SPSS (SPSS Inc., Chicago, IL, USA) for statistical analysis. A one-tailed t-test (α = 0.05) was used to compare the percentage of PNNs associated with PV+ cells and the reported proportion of PV+ interneurons in the striatum. To obtain a better understanding of the relationship between striatal PNNs and PV+ neurons, two overlap ratios were calculated: The percentage of total PNNs associated with PV+ cells, and the proportion of PV+ cells enclosed by PNNs. A repeated measures ANOVA (within-subject comparison across three axes (factors): rostrocaudal (3 corresponding to the three sections), dorsoventral (dorsal (D) vs. ventral (V) halves of each section were divided by a horizontal line halfway between the dorsal and ventral extremes of the striatal reconstruction) and mediolateral (medial (M) vs. lateral (L) halves bisected by a vertical line halfway between the mediolateral extremes of the striatal reconstruction); α = 0.05), was used to compare the regional densities of PNNs and PV+ cells, as well as the extent of overlap between PNNs and/or PV+ cells (for both values).

### Chondroitinase ABC injections

To ascertain the degree and time course of enzymatic PNN digestion, adult male mice (8–11 months old) received unilateral injections of chondroitinase ABC (ChABC) (protease free; 15 U/mL in 0.9% saline; Sigma Aldrich, St. Louis, MO) within the striatum as described previously [25]. Briefly, animals were anesthetized with 2–4% isoflurane in oxygen and placed in a mouse stereotax (Kopf Instruments, Tujunga, CA, USA). Injections were made 2 mm directly lateral to bregma, at a depth of 2 mm using heat-pulled glass micropipettes connected to a picospritzer (WPI, Sarasota, FL, USA). At 3 (n = 3) and 14 days (n = 3) after ChABC treatment, mice were euthanized and brains harvested, then processed for WFA labeling as described above.

In mice undergoing behavioral testing, either ChABC or vehicle (0.9% saline) was injected bilaterally in the striatum as described above. Animals were then allowed to recover for 3 days before task onset. To both confirm the extent and localization of striatal PNN digestion by ChABC and to assess the state of saline-injected striatum, brains from all treated mice were harvested, sectioned, processed for WFA binding (see above), and imaged using a deconvolution microscope (Zeiss) with a 5× objective (N.A. 0.16). For three representative animals, the extent of digestion was further characterized by comparing the size of the PNN free zone with total striatal area in coronal sections corresponding to the injection site (n = 5 hemispheres).

### Behavior

#### Gait measurements

In order to assess the gait of adult (8–11 months old) mice that received chondroitinase (n = 11) or saline (n = 9) injections, step width and variance was measured and compared between groups. Briefly, on the third day after bilateral injections, individual animals had petroleum jelly applied to both hind paws and were placed on a paper track in a rectangular box (dimensions (length×width×height): 60 cm×16 cm×30 cm). Subjects were allowed to explore the enclosure until they walked from one end of the paper track to the other. The mice were then removed, and graphite powder (Sigma Aldrich) was sprinkled on top of the paper to visualize pawprints. Gait parameters were measured as described previously [65]. Briefly, step width was defined as the perpendicular distance from a given paw print (specifically, the point between the two middle pads of the paw) to a line joining two consecutive pawprints of the opposite hind limb. Variance is defined as the standard deviation of step widths squared for a given animal. Sets of paw prints were only considered for analysis if there were 8 or more consecutive steps without a change in direction. A Wilcoxon rank sum test was used to compare measurements between ChABC-treated and saline-injected mice.

#### Morris water maze

On the fourth day following gait assessment, ChABC treated (n = 9), and saline injected mice (n = 9) were further assessed on MWM performance as described previously [27]. Briefly, mice were gently placed in one of four designated starting points within four equally sized quadrants (A, B, C, D; see inset Fig. 4B) on the periphery of a large pool (diameter: 91 cm) filled with opaque water (depth: 19.5 cm) at room temperature (23.5 to 25°C). Animals were monitored for their ability to acquire the location of an escape platform (diameter: 11.5 cm) submerged ∼1 cm below the waterline across 7 consecutive days. Each day, individual mice had to complete four trials from each of the four starting points presented in a different order which was varied day to day. Trials ended when animals reached and remained on the platform for 10 seconds. On any given trial, subjects were removed from the pool if they failed to discover the platform within 60 seconds. On the eighth day, all mice were exposed to a single probe trial (platform removed) in order to assess their ability to recall the acquired platform location. A Quickcam digital video camera (Logitech, Fremont, CA, USA) mounted vertically above the pool was used to monitor and record behavior. After completion of the last trial, animals were immediately euthanized, brains extracted and WFA-lectin binding was performed as described above. All behavioral tasks were completed within 14 days after receiving striatal injections.

#### Analysis

Two measurements were used to assess differences in task acquisition between treatment groups: Latency (time to find the escape platform from the starting position, or 60 seconds on failure, whichever occurred first) and distance (the total length of swim trajectory). Both measurements were extracted from videos acquired during training using Image J (NIH). Average swim speed was calculated as total distance traveled to reach the escape platform divided by latency. A repeated measures ANOVA (group as the between-subject factor, and latency or distance across days as the within-subject repeated measure) was used to reveal performance differences between ChABC and vehicle treated animals (α = 0.05) for the duration of the 7 day task acquisition. This procedure was followed by a one-way ANOVA, comparing groups (ChABC vs. vehicle) at each level of the repeated measure (day). For the probe, a repeated measures ANOVA (group as the between-subject factor, and quadrant as the within-subject repeated measure) was used to assess group differences in terms of time spent in the platform quadrant.

## Supporting Information

Figure S1 **PNNs show no overlap with either ChAT+ or Cr+ interneurons in the striatum.** (A) Panels reveal WFA/PNN labeling (left), ChAT+ (middle) immuno-staining, and a merged image of the two (right). No overlap was observed between WFA/PNN (arrows) and ChAT+ cells (arrowheads). (B) Similarly, no Cr+ cells (arrowheads) were seen to be ensheathed by PNNs (arrows). Panel conventions are identical to A. Scale bar: 100 µm.
(TIF)

Figure S2 **ChABC digested striatal PNNs show no regrowth 14 days following treatment.** A) Untreated control hemisphere. B) ChABC treated hemisphere. PNNs are present in control but not ChABC treated side. Note PNNs are present in cortex and surrounding structures of the ChABC treated hemisphere, indicating that the digestion is localized to the striatum. Scale bar: 1 mm.
(TIF)
