# Supplementary figures and images for: Perineuronal Nets Play a Role in Regulating Striatal Function in the Mouse
Source: PLoS One. 2012 Mar 12;7(3):e32747. doi: 10.1371/journal.pone.0032747 (PMC3299692; doi:10.1371/journal.pone.0032747)

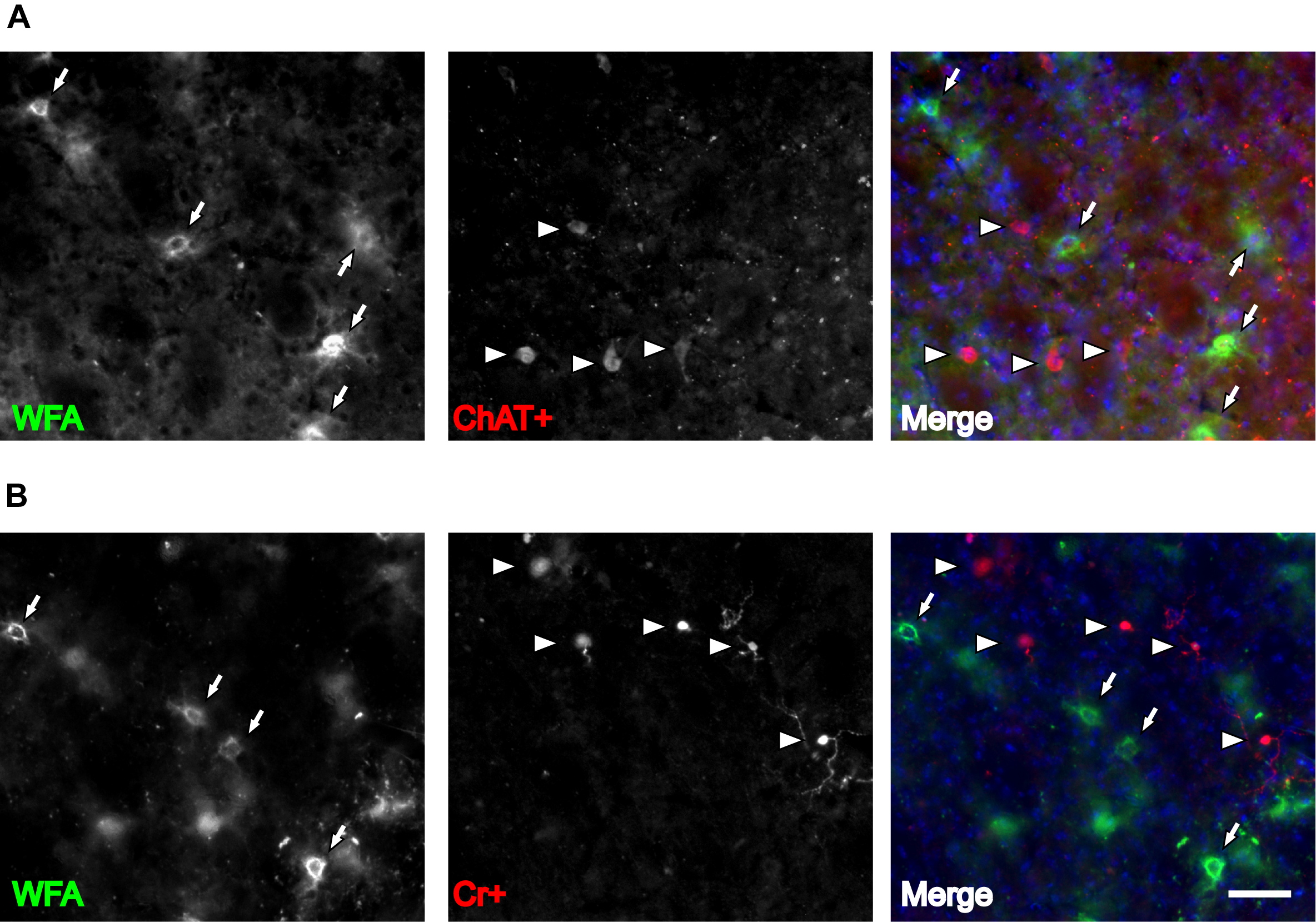

Supplement: Figure S1 — PNNs show no overlap with either ChAT+ or Cr+ interneurons in the striatum. (A) Panels reveal WFA/PNN labeling (left), ChAT+ (middle) immuno-staining, and a merged image of the two (right). No overlap was observed between WFA/PNN (arrows) and ChAT+ cells (arrowheads). (B) Similarly, no Cr+ cells (arrowheads) were seen to be ensheathed by PNNs (arrows). Panel conventions are identical to A. Scale bar: 100 µm. (TIF) [file pone.0032747.s001.tif]

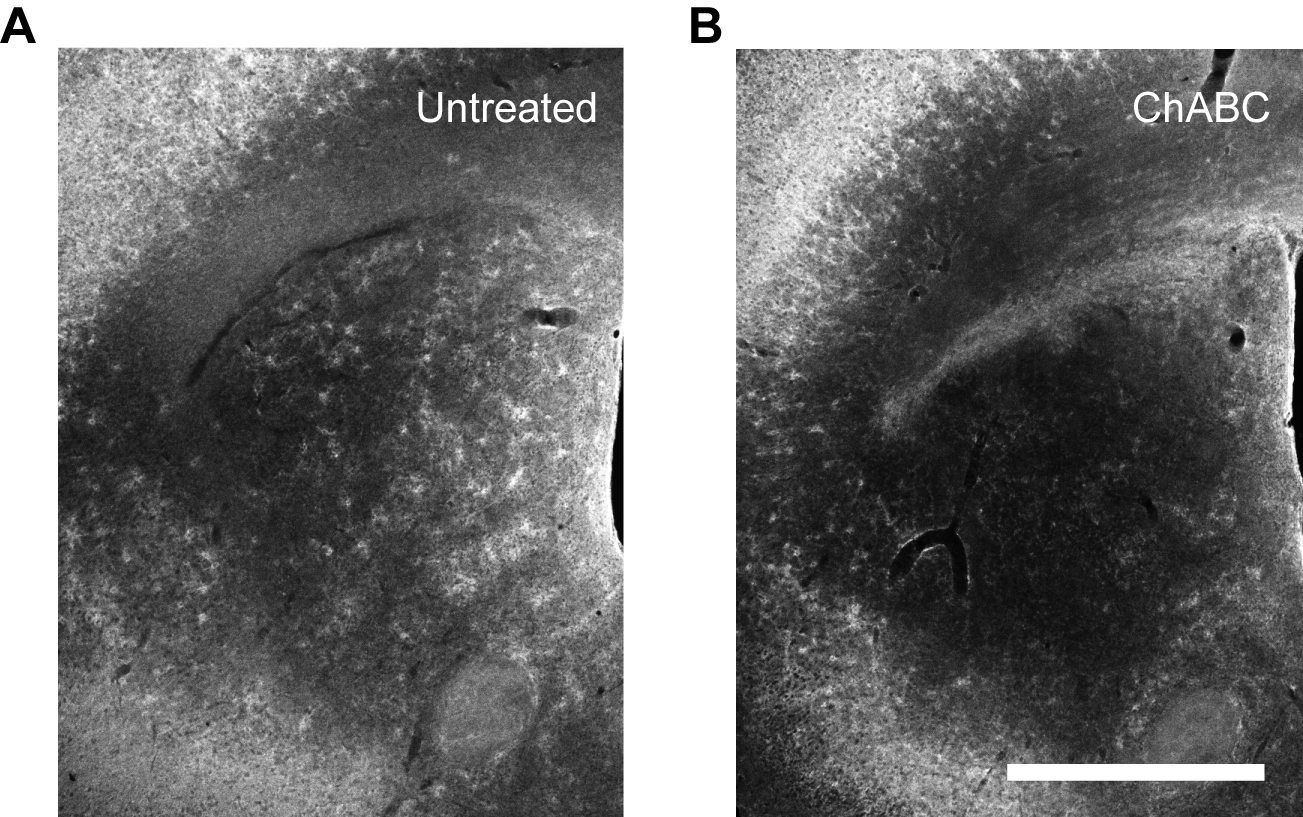

Supplement: Figure S2 — ChABC digested striatal PNNs show no regrowth 14 days following treatment. A) Untreated control hemisphere. B) ChABC treated hemisphere. PNNs are present in control but not ChABC treated side. Note PNNs are present in cortex and surrounding structures of the ChABC treated hemisphere, indicating that the digestion is localized to the striatum. Scale bar: 1 mm. (TIF) [file pone.0032747.s002.tif]
